# Perceived discrimination in fatigue: a qualitative interview study in the SOMA.SOC project

**DOI:** 10.3389/fsoc.2025.1528312

**Published:** 2025-02-05

**Authors:** Rieke Barbek, Anna Christin Makowski, Olaf von dem Knesebeck

**Affiliations:** Institute of Medical Sociology, University Medical Center Hamburg-Eppendorf, Hamburg, Germany

**Keywords:** fatigue, intersectionality, intersectional discrimination, intersectional stigma, perceived discrimination, social identities

## Abstract

**Introduction:**

Fatigue is a frequent somatic symptom impacting health and well-being and lately receiving increased attention as a long-term consequence of COVID-19. Emerging evidence suggests that persons afflicted with fatigue symptoms are often stigmatized and discriminated because their symptoms are still poorly understood and not recognizable to others. Existing stigma research mainly focused on specific medical conditions and domains and overlooked intersectional discrimination – the negative amplification effect of intersecting social identities. The purpose of the current study is to examine perceived discrimination in fatigue across different medical conditions and domains, also considering intersectional discrimination.

**Materials and methods:**

Semi-structured telephone interviews were carried out with 19 patients with clinically significant fatigue, considering a variety of different social identities like gender, history of migration, and occupational status. The interviews were analyzed using a structured qualitative content approach with consensual coding.

**Results:**

The findings on perceived discrimination could be subsumed in eight practices: (nonverbal) communication, negative emotional reaction, medical treatment, leadership responsibility, structural barriers, diagnostic terminology, and scientific controversy. Participants reported overlapping experiences of perceived discrimination across several intertwined domains: medical setting, work, social, public, and at an overarching structural level. Thereby, especially discrimination in the medical setting and on structural level occurred with great impact on health care and social protection. By applying an intersectional approach, intersectional discrimination specific for certain stigmatized social identities, like female gender and low occupational status became apparent.

**Discussion:**

These findings need to be further researched and addressed in intervention strategies increasing resilience and public knowledge to reduce intersectional discrimination and health inequalities.

## Introduction

Stigmatization is understood as a complex social process that profoundly impacts individuals, particularly those affected by mental disorders, (chronic) diseases, or otherwise stigmatized social identities (Major et al., 2018). Link and Phelan (2001) have conceptualized a model of the stigma process that has been frequently used in the associated research. Accordingly, stigma is a multidimensional construct in which several interrelated components are present: labeling (perceived deviations from social norms are labeled as “different”), stereotyping (prevailing cultural norms link these labels to stereotypes) and separation (from “us” and “them”). This process comes into effect in relationships and interactions that are characterized by an imbalance of power. As a consequence, a person is placed further downward in social hierarchy and experiences status loss and discrimination (Link and Phelan, 2001). In this regard, discrimination is the last (behavioral) component of the stigma process.

Expanding on this work, Thornicroft et al. (2022) further elaborated on the pervasive impact of stigma across different domains of society – from institutional policies to interpersonal interactions. The authors advocated for a social-ecological approach, recognizing that stigma operates at multiple levels, and can therefore have an impact on those affected in different domains of their lives: in their circle of family and friends, at work, in health care, in society in general, or at an overarching structural level, for example when dealing with authorities or health insurance companies. This approach provides a comprehensive framework for understanding the full spectrum of stigma and informs strategies to mitigate its harmful effects (Thornicroft et al., 2022).

In this study, we refer to perceived discrimination, i.e., the perception of being devalued and discriminated against by others. Perceived discrimination imposes a great burden on those affected with negative consequences for their health, quality of life, self-efficacy in various areas of life, social participation, ability to work, health care, and social protection (Link and Phelan, 2001; Stangl et al., 2019). Moreover, the fear of social stigma or perceived discrimination can be a barrier that impedes diagnosis of several somatic, mental and somatoform disorders (Marinho and Barreira, 2013; Murray et al., 2016; Sims et al., 2021).

Research on health-related stigma often lacks other social identities that also can be associated with stigmatization, e.g., gender minority status, low socio-economic status, or ethnic minority (Bowleg, 2012). For example, women are often affected by sexism and gender-based violence, whereas men are confronted with exaggerated images of masculinity. People with a history of migration are often affected by (cultural) racism and other negative experiences pre-, during and post-migration. A low socio-economic status is often associated with experiences of classism and limited resources for fulfilling life chances (Bowleg, 2012; Courtenay, 2000). Common to all -isms is increased distress with further negative consequences for health, but also – at least to some extent – resilience and political power. The intersection of these social conditions is addressed in the concept of intersectionality (Bowleg, 2012; Crenshaw, 1989). Only recently, the two concepts of intersectionality and health-related stigma/discrimination were merged in the concept of intersectional stigma or intersectional discrimination, respectively (Council of Europe, 2024; Turan et al., 2019). This implies the convergence of multiple stigmatized social identities within a person or group, which results in joint or cumulative effects regarding health outcome and associated consequences. Research on intersectional stigma initially focused on HIV and gender/sexual minorities. Findings are mixed and research needs to be expanded on further social identities and health outcomes (Henkel et al., 2008; Pescosolido and Martin, 2015; Turan et al., 2019).

Persistent somatic symptoms recently became a focus of health research. Many somatic symptoms cannot exclusively be ascribed to either a somatic disease, e.g., cancer, or a mental disorder, e.g., depression (Löwe et al., 2022). Therefore, the dualistic view of either a somatic or a psychological causation seems inappropriate. Rather, a biopsychosocial approach is needed. Persistent somatic symptoms are used as an umbrella term to describe subjectively distressing somatic complaints, irrespective of their etiology, that are present on most days for at least several months (Löwe et al., 2024). Persistent somatic symptoms cause substantial suffering, stigmatization, and impaired quality of life and work participation (Joustra et al., 2015; Ko et al., 2022; Löwe et al., 2024).

A frequently occurring persistent somatic symptom is fatigue, also described as exhaustion or tiredness, with a wide spectrum of shapes and sizes (Olson et al., 2015). Among the complaints with the greatest impact on health and well-being, fatigue ranks in 2nd place behind pain (Krabbe et al., 2019). The prevalence of fatigue symptomatology has been estimated at approximately 30% in the adult population in Germany (Kocalevent et al., 2011), whereat the prevalence of “unexplained fatigue syndromes” varies internationally between 2 and 15% (Skapinakis et al., 2003). As a concomitant symptom, fatigue can occur in different somatic and mental disorders like cancer, multiple sclerosis, or depression (Baum et al., 2022; Stuke et al., 2009). As a leading symptom of extreme severity, fatigue appears in the neurological multisystem disease myalgic encephalomyelitis/chronic fatigue syndrome (ME/CFS) which might be caused by a previous viral or bacterial infection, among others (Baum et al., 2022). Fatigue symptomatology can also occur in long COVID in its most severe form as ME/CFS (Global Burden of Disease Long COVID Collaborators et al., 2022; Koczulla et al., 2024). The etiological mechanisms of fatigue as a persistent somatic symptom are still not sufficiently understood (Baum et al., 2022; Koczulla et al., 2024; World Health Organization, 2024). Fatigue is more frequently reported by women, younger people, and people of low socio-economic status (Clutterbuck et al., 2024; Engberg et al., 2017; Watt et al., 2000). Regarding history of migration, evidence is lacking. However, research indicates higher prevalence of, e.g., ME/CFS in communities of people of color linked to the concept of cumulative stress burden (Fennell et al., 2021).

Emerging evidence suggests that persons afflicted with fatigue symptoms are often stigmatized and discriminated because their symptoms are still poorly understood, often not recognizable to others, unacknowledged, and psychologized by health professionals and the public (Asbring and Närvänen, 2002; Clutterbuck et al., 2024; König et al., 2024). In severe cases like ME/CFS, discrimination can even be experienced as a heavier burden than the disease itself (Asbring and Närvänen, 2002). However, fatigue seems less stigmatized when it occurs in the context of a widely accepted (somatic) disease such as multiple sclerosis (Ko et al., 2022; McInnis et al., 2015). In contrast, the less a disease is known and accepted, e.g., in case of ME/CFS or long COVID as a quite new phenomenon, the more the disease and the people affected might be stigmatized and discriminated (Brehon et al., 2023; Moretti et al., 2022). Regarding intersectional discrimination in fatigue, evidence is lacking. A recent study documented multiple public stigma in fatigue regarding gender, history of migration, and occupational status (von dem Knesebeck and Barbek, 2023).

There is growing evidence of perceived discrimination in specific medical conditions related to fatigue, like ME/CFS or long COVID. However, research is lacking the focus on fatigue as a persistent somatic symptom independent of any underlying disease. In addition, existing stigma research mainly overlooks the potential negative amplification effect of intersectional discrimination. Accordingly, the current study addresses the following research questions: (1) In relation to their symptomatology, what perceived experiences of discrimination do patients with fatigue encounter and in which domains do they take place? (2) Do potentially stigmatized social identities like gender, history of migration, or occupational status intersect with health-related perceived discrimination in patients with fatigue?

## Materials and methods

### Study design

The current study is part of a project on social inequalities in aggravating factors of persistent somatic symptoms (SOMA.SOC), which is embedded in the Research Unit 5211 “Persistent SOMAtic Symptoms ACROSS Diseases: From Risk Factors to Modification (SOMACROSS)” (Löwe et al., 2022; von dem Knesebeck et al., 2023). Two persistent somatic symptoms were focused with high prevalence in Germany: fatigue with a prevalence rate about 30% (Baum et al., 2022) and irritable bowel syndrome (IBS) with prevalence rate about 10–20% (Layer et al., 2021). The project collected both quantitative and qualitative data. The vignette-based quantitative telephone survey conducted in Germany is described in detail in the study protocol (von dem Knesebeck et al., 2023). For the qualitative study part, longitudinal interviews were held with patients affected by fatigue or IBS, respectively, at three points in time (baseline and after six and 12 months). To investigate social inequalities and to obtain a variety of experiences, participants were stratified according to the following social identities: gender (male/female), occupational status (high/low), and history of migration (yes/no). Regarding gender, people who specified their gender as diverse were excluded from the study. With respect to the occupational status of participants, the International Socio-Economic Index of Occupational Status (ISEI), according to Ganzeboom et al. (1992), was used as a criterion for assigning scores (Ganzeboom et al., 1992). The ISEI index refers to the International Standard Classification of Occupations (ISCO-08) (International Labour Organization, 2024). Scores were dichotomized to obtain groups of high and low occupational position (Lampert et al., 2013). Regarding the participants’ history of migration, the definition of the German Federal Statistical Office was adapted to cover people who immigrated themselves or have at least one immigrated parent (Bartig et al., 2023). As the current study considered only baseline interviews of patients with fatigue, solely details regarding the fatigue sample are provided in the following. For details concerning the IBS sample, please refer to the study protocol (von dem Knesebeck et al., 2023).

For recruitment of participants, following purposive sampling, a multi-stage process was established. First, a collaborating physician contacted the patients with fatigue listed in the patient register of a general medical outpatient clinic by telephone. Subject to patient’s consent, patients were again contacted by a member of the study team to provide further study information and to check for inclusion (the inclusion and exclusion criteria are described below). The collaborating physician received 50€ per included patient as expense allowance. Second, a multitude of (medical) institutions were contacted via telephone and/or email to inform about the study. Relevant institutions were searched for as follows: general practitioners, medical specialists, polyclinics, and private clinics either involved in the medical care of patients with fatigue or located in neighborhoods with a relevant proportion of people with a history of migration which were listed in a doctor search engine of the Hamburg association of statutory health insurance physicians; advisory centres and psychotherapists with a focus on the subject of migration. Supportive institutions either placed leaflets on their reception counters or emailed the leaflet to their patients. Interested patients could then contact the study team by phone, email, or participate (via QR-code) in an online survey to receive further study information and be checked for inclusion (the inclusion and exclusion criteria are described below). If eligible, a study information package including a written informed consent form was sent to the patient by post. Participants were first recruited in Hamburg and the surrounding region. Later on, it was extended to northern and central Germany, as some specialized doctors had a correspondingly large catchment area. After each conducted interview, 20€ were payed to the participants as expense allowance.

The following inclusion criteria were defined: age ≥18 years, sufficient spoken and written German language proficiency, and written informed consent. Furthermore, either a ICD-10 diagnosis for fatigue as determined by a general practitioner or chronic fatigue assessed via the Fatigue Scale with a cut-off for clinical relevant chronic fatigue ≥4 points (Martin et al., 2010), in combination with at least one medical consultation, was needed. Exclusion criteria were: serious illness requiring immediate intervention, florid psychosis or substance abuse disorder, and acute suicidality. Additionally, for a balanced sample according to health condition, sex, history of migration, and occupational status, people were considered non-eligible when they did not meet the stratification criteria.

### Data collection

Enrollment of participants and conducting baseline interviews lasted from January 2023 until January 2024. Data collection continued until the aim of at least two baseline interviews per stratum (condition x gender x occupational status x history of migration) was reached. The only exception were males with a history of migration. Even with extended recruitment efforts, not enough suitable participants could be enrolled in the study. Nevertheless, the sample size of *n* = 19 participants was larger than the minimum sample size (12 interviews) suggested for qualitative studies (Braun and Clarke, 2013; Guest et al., 2006). During the recruitment phase, *n* = 170 interested persons were not eligible for participation due to various reasons, e.g., refusal of consent to data protection for the online survey (64.7%), diagnostic criteria not fulfilled (14.7%), or stratification criteria not fulfilled (15.3%).

Interviews were conducted by telephone using a semi-structured interview guide with open-ended questions (see Table 1). Follow-up questions further specified mentioned topics. The precise formulation and order of questions could be varied (Adams, 2015). This allowed to cover the main issues with all participants while also offering flexibility in discussing issues pertinent to individuals. The interview guide was pre-tested both with a volunteer patient who was not included in the study and with colleagues of the research team. This process identified necessary modifications that could then be implemented directly in the initial interview guide (i.e., redundancies or needs for specification). Interviews were audio-recorded upon participant’s verbal permission. Interviews were transcribed using the freeware noScribe as artificial intelligence technology for automated audio transcription (https://github.com/kaixxx/noScribe, accessed Dec 12th, 2023). The automatically generated transcripts were edited by two project team members against pre-established uniform transcription rules using the transcription software f4 (https://www.audiotranskription.de). The length of the interviews varied between 31 and 77 min, with a mean of 46 min.

**Table 1:** Topics of the interview guide.

Topics	Specification
(1) Origin, causes and development of the symptoms	Symptom perception, course and severity of symptoms, health anxiety
(2) Coping and help-seeking	Behavior and experiences in dealing with symptoms, health literacy, treatment experiences, treatment expectations
(3) Social interaction and perception by others	Disclosure of symptoms, public stigma and perceived discrimination associated with the symptoms

### Data analysis

For the analysis of the transcribed interviews, structured qualitative content analysis according to Kuckartz (2018) and Kuckartz and Rädiker (2020) was used. A core aspect of this approach is to make the interpretation of text describable and verifiable. The approach follows an iterative process consisting of text work, categorization, coding, analysis and presentation of results. A deductive-inductive coding system was specifically suitable to answer the first research questions. Thereby, domains of perceived discrimination were defined as deductive categories (with optional addition of any other sources as inductive categories) based on Thornicroft et al. (2022), while the reported experiences were derived from the material as inductive main categories (main practices of perceived discrimination) and further specified in subcategories (sub practices of perceived discrimination). The consensual coding process was carried out by two coders to enhance intersubjective comprehensibility (Kuckartz, 2018). Therefore, the two coders independently created a coding system on the basis of pre-selected transcripts and determined the appropriate code segments. Findings were then consented regarding consistency and precision, e.g., semantic exclusiveness of the subcategories. This collaborative coding process took place until inter-coder agreement was reached across all the interviews. The final code system was then applied to all interviews by the two researchers. Findings were compared and again consented, where necessary. As a central technique within qualitative research processes, memo writing was crucial in the form of coding rule memos. In addition, code summaries were written to condense findings. Based on the code summaries, fatigue-related perceived discrimination was extracted. The extracted experiences were further compared across individuals according to their social identities to answer the second research question. To illustrate the experiences of the participants, a variety of quotes can be found in the results section. These are provided with a specific key representing the social identities of the respective participant: F female, M male, hO high occupational status, lO low occupational status, nMh no history of migration, Mh history of migration.

The iterative analysis process was performed with MAXQDA 24 software (Kuckartz, 2018), which allows for continuous analytical reflection throughout the qualitative research process.

### Rigor

The validity of the study was investigated according to Lincoln and Guba’s criteria, consisting of credibility, transferability, dependability, and confirmability (Lincoln and Guba, 1985). Credibility was ensured through prolonged engagement, selection of participants with different social identities, persistent interviewing over a one-year time period, analyst triangulation with two researchers carrying out the interviews and three researchers reviewing the findings, and negative and deviant case analysis. Maximum variation sampling, accurate description of the participants, the sampling method, and the time and place of data collection increased the transferability of the data. To facilitate dependability, all members of the research team contributed to coding the interviews and/or data analysis, and findings were further discussed with colleagues of the research unit. Confirmability was established by providing a rich description of the data to enable evaluation by external observers and obtain a clear understanding of the study procedure. Also, the members of researcher team continually reflected the potential impact of their own position, perspective, beliefs, and values throughout the research process.

### Ethical considerations

The study was planned and carried out following the principles of the Declaration of Helsinki and the European Data Protection Law. The study design was approved by the Ethics Commission of the Hamburg Medical Chamber (No. 2020-10194-BO-ff). Considering the potentially emotional nature of the interviews, participants were assured that their information would be treated confidentially and that consent could be withdrawn at any time without negative consequences. In case of participants experiencing severe psychological distress during the interview, the Outpatients Clinic for Psychosomatic Medicine and Psychotherapy at the University Medical Center Hamburg-Eppendorf could be contacted by telephone. In publications, participants’ data and quotations are reported anonymously.

## Results

### Study sample

Through purposive sampling, a study sample including *n* = 19 participants with different social identities was realized (see Table 2). Approximately half of the sample consisted of women and of people with high occupational status. Two thirds had no history of migration and were aged 40+ years. Concerning symptom specifics, one fourth of participants stated that they had not yet received a diagnosis for their fatigue symptomatology. If diagnoses were made, according to patient statements, these were ME/CFS, fatigue (not specified), and long/post COVID. The onset of symptoms ranged from childhood to less than 5 years ago. The majority of participants was on sick leave or received temporary disability or sickness pension. Only a few were employed full-time or part-time.

**Table 2:** Participant characteristics (*n* = 19).

	*n*	%
Gender		
Female	11	57.9
Male	8	42.1
Age		
<40	6	31.6
40+	13	68.4
Range	30–69	
History of migration		
No	13	68.4
Yes	6	31.6
1st generation	1	16.7
2nd generation	5	83.3
Occupational status		
High	10	52.6
Low	9	47.4
Employment		
Yes (including reduced working hours)	7	36.8
No (including sick leave, early retired, partly/temporary disabled)	11	57.9
n.s.	1	5.3
Diagnosis*		
No	7	36.8
Yes	12	63.2
ME/CFS	6	50.0
Fatigue (n.s.)	4	33.3
Long/post COVID	2	16.7
Onset of symptoms		
<5 years	7	36.8
5–10 years	4	21.1
Childhood/adolescence	6	31.6
n.s.	2	10.5

ME/CFS, Myalgic encephalomyelitis/chronic fatigue syndrome; n.s., not specified.

*As stated by the participants.

### Practices of perceived discrimination

Qualitative data analysis resulted in eight main practices, each with several corresponding sub practices of perceived discrimination (see Table 3). The deductively selected domains in which discrimination was perceived by participants followed Thornicroft et al. (2022). No further inductive domains were extracted from the interviews. Accordingly, participants reported perceived discrimination particularly in the medical setting. Beyond that, work-related discrimination as well as perceived discrimination in the social environment, especially in contact with family and friends, occurred. Some participants further elaborated on perceived discrimination in public, in the media, or at a structural level including science and research.

**Table 3:** Practices of perceived discrimination in patients with fatigue (*n* = 19).

Main practices	Sub practices
#1 Communication	Trivialize Relativize Devaluation Negation Ignorance Psychologization Sexism Reduction to outer appearance Put under pressure Impose excessive help
#2 Nonverbal communication	Smile things away Stare Rejection Turn away
#3 Negative emotional reaction	Anger
#4 Medical treatment	Lack of engagement with symptom pattern (lack of time/willingness) Withhold diagnostics/misdiagnosis against/without medical findings ~ Psychologization Refusal of treatment/responsibility Assertion of lack of treatment options Counterproductive advice (e.g., medication, physical activity) Threaten drastic consequences Ignorance of patients’ special needs Lack of coordination between doctors is blamed on patients
#5 Leadership responsibility	Withdrawal of day-to-day business Refusal of safety precautions/reintegration measures Dismissal due to sick leave
#6 Structural barriers	Bureaucracy outranks best possible treatment Lacking scope of services Ignorance of symptom specifics Barriers of access
#7 Diagnostic terminology	Diagnostic terminology plays down severity of symptoms
#8 Scientific controversy	Ongoing controversy regarding etiology, neglect of neurological classification

#### Medical setting

Particularly in the medical setting, participants reported a number of experiences that they perceived as discriminatory and stigmatizing regarding their medical treatment (see #4 Table 3). During initial consultations, but also during follow-up contacts, patients experienced that some doctors stated that they could not spare the time to deal with *“something like this”* (*M/hO/nMh*), and felt dismissed. Even more drastic were consultations in which the practitioners made it clear that they simply refused to deal with the complex issue in more detail and transferred this responsibility to the participant.

*“My doctor is all about that, just his budget. He does not care what happens to a person. Even with my illness, he said it’s a new disease, it takes a long time to research it. […] If I asked any questions, he said, no, I do not know. Do your own research.” (F/lO/Mh).*

Also, in their efforts to obtain a diagnosis for their symptoms, some of the participants had the impression of being turned down or being misdiagnosed in their own terms. In particular, psychologization should be mentioned here, which was rejected by most participants as it was based on rather insufficient arguments such as *“There is no biomarker, so it must be psychological.” (M/hO/nMh)* or *“It is just another form of depression.” (M/hO/Mh)*.

Further descriptions of interactions in the medical setting from the participants’ perspective could be placed gradually. They ranged from a rejection of treatment responsibility on the grounds that *“they [RB: the neurologists] are not the right people”* (*F/lO/nMh*) to a clear refusal of treatment with the words *“The doctor opened the door, such a disease does not exist. Goodbye.” (F/lO/Mh)*. In some cases, treatment was initiated, but resulted in advice that the patients rated more harmful than beneficial.

*“I need to get my immune system back on track with iron and vitamin D and otherwise it’s a case of wait and see. That was the medical advice so far, I would have to eat more meat to improve my iron levels and then all this tiredness and everything would just go away.” (F/lO/nMh)*.

For one participant, her stay in a rehabilitation facility turned into an extremely stressful experience. Her description revealed various aspects that other participants also reported, namely the – sometimes implicit – accusation of not belonging there or *“not being right”* (*F/lO/nMh*) in this medical specialty, or the psychologization already described. In this particular case, the participant was also threatened with drastic consequences. Strong negative emotional reactions such as anger became apparent in this behavior (see #3 Table 3).

*“So it’s super frustrating and especially as a young woman, it’s also quite a lot to deal with, including rehab. The consultant, she did not know anything about the disease, and I had a crash in rehab where nothing really worked. And then she shouted at me and said that from her point of view, I was severely depressed and I had to pull myself together now, otherwise I’d be going home tomorrow. I just started crying, how am I supposed to get home, because I could not move at all.” (F/hO/Mh).*

Participants also reported perceived discrimination in the medical setting with respect to communication (see #1 Table 3). The participants were confronted with statements ranging from trivialization like *“It will get better on its own.” (F/lO/nMh)* and relativization, e.g., *“everyone gets tired and everyone gets exhausted” (M/hO/nMh),* to even personal devaluation and negation, often in combination with a psychologization of the symptoms, which was in their eyes unjustified.

*“And everyone always tells me, well, CFS, if you are happy with the diagnosis, then be that, but the illness does not exist. It’s all because of your depression. And that’s actually how every conversation went with all the doctors besides Professor X [RB: head of a private clinic]. And it’s so awful, I get tears in my eyes again just thinking about that time. It was actually horror.” (M/hO/nMh).*

Especially (younger) women further described sequences of conversations that were characterized by sexism and reduction to outer appearance, again often paired with psychologization. The interviews repeatedly contained statements such as *“He [RB: the doctor] totally dismissed it, he said you are still young, you look good.”* (*F/lO/nMh*).

In addition to actively expressed forms of perceived discrimination, one participant also reported being smiled away as part of nonverbal communication (see #2 Table 3): *“I had the feeling, especially with my neurologist, that he did not take me seriously. He kind of smiled it away.” (F/hO/Mh)*. One participant further described negative emotional reactions of healthcare staff when it was confronted with the symptoms, which are not always visible from the outside.

*“And you probably know this yourself, when you go to the doctor […] a doctor’s assistant takes you somewhere. They run like there’s no tomorrow. You cannot keep up with them. And if you, as a fit-looking person, shuffle along slowly, they almost get annoyed.” (M/hO/nMh).*

#### Work

On the one hand, participants reported work-related discrimination in the direct interaction and (nonverbal) communication with colleagues (see #1 and #2 Table 3). The symptoms were psychologized, for example misjudged as a burnout. Also repeated comments were made about the outer appearance. Either because the participants looked *“very good” (F/lO/Mh)* and supposedly healthy and then were put under pressure to return to work. Or, if the exhaustion was recognizable, the colleagues sought for reasons that corresponded to their own world of experience, as the symptoms were not comprehensible to them, so mentioned by another participant. In other cases, participants’ symptoms were ignored and colleagues turned away from participants.

*“They noticed, look at her, yes, you have had a long night, you have been partying, because of course I then had rims under my eyes because I could not sleep well or the fatigue was exhausting. I suffered from that.” (F/lO/nMh).*

On the other hand, the participants described discriminatory behavior of their superiors (see #5 Table 3). This was expressed, for example, in the fact that reintegration into the company was taken up only hesitantly or not at all, although there are nationwide guidelines on how to proceed in these cases and ensure a successful comeback to your workplace after a longer period of illness.

*“I was on sick leave in the middle of May last year, then did rehab at the end of the year, then wanted to start again in January. My boss did not react at all to my reintegration. Not at all. He did not want me back. Then I obtained a health certificate in May [RB: the following year] and was then released from work for eight weeks and transferred to another department, where I’ve had nothing to do ever since.” (F/hO/Mh).*

The same participant then went on to say that she had experienced an absolute withdrawal of tasks and responsibility by her superiors. And although she was in touch with her union representative as well as the head of human resources several times, no consequences have arisen so far, making this *“a really extreme situation”* (*F/hO/Mh*) for her. In other cases, participants reported that they had been dismissed. They did not necessarily attribute this to the symptoms per se, but rather to the duration of the respective sick leave. However, this necessary duration is in turn inextricably linked to the complexity of the symptomatology.

*“I think it was more the duration. Because it wasn’t foreseeable when I would get well again. And I think I would have just been an administrative burden. Or a factor that was difficult to plan. That’s why it was easier to dismiss me. But not necessarily because of the nature of the illness.” (M/lO/nMh).*

#### Social environment

Regarding the social environment, perceived discrimination was described in particular in the course of communicating with family and/or friends (see #1 Table 3). Here, too, especially female participants reported different forms of discrimination, like relativization, trivialization and negation, partially influenced by culture. *“She [RB: patient’s sister] says that there is no such disease in Turkey. Or about forgetfulness: Oh, I forget too, we are too old. I cannot do math either.” (F/lO/Mh).* Participants also reported being reduced to their outer appearance by their family and/or friends and being confronted with incomprehension: *“My family too. But you look good. Why? Yes, why do not you do it!” (F/lO/Mh).* Participants also reported of being put under pressure to do things or to get active, like *“just get out and go for a walk” (F/lO/nMh)*. Besides, participants also stated that their social environment sometimes imposed excessive help on them: *“They sometimes make it difficult in that sense, because then they try to help and then they mean it too well, because then they get in touch every day.” (F/lO/nMh).* A male participant further explained that he no longer wanted to be exposed to the social pressure of having to recover soon: *“So I completely put a lot of acquaintances aside. Because then you have phone calls, they mean well and then say, yes man, that has to get better again.” (M/hO/nMh).*

#### Public

In some cases, participants also reported negative experiences in the public (see #1 Table 3). These relate to the negation or psychologization of the symptoms. In particular, some repeated comments in the media were perceived as highly discriminatory and sexist. The acceptance of the symptoms as a disease was also repeatedly questioned, reported by a participant with long COVID. Participants further described experiences of nonverbal discrimination (see #2 Table 3) in the form of being stared at or rejected, as well as negative emotional reactions, e.g., one participant reported his experience of the public often not knowing how to properly deal with the symptoms when confronted.

*“Then I start slurring my words and people think I’m drunk. And I’ve already had such bad situations, I do not want that again.” (M/hO/nMh).*

#### Structural level

Looking at the bigger picture, it can be seen that some of the distressing experiences that those affected have to go through are rooted in structural conditions that are difficult to overcome (see #6 Table 3). In some cases, participants were left with the impression that compliance with bureaucratic processes is more important than providing the best possible care for those affected.

*“There were some [RB: rehab facility with specialization/focus on long COVID], but they were just overcrowded. So, I think the pension fund just wanted to send me somewhere so that I would be considered fit for work again afterwards, and that was it.” (F/hO/Mh).*

Even if this is possibly born out of a lack of capacity, participants repeatedly reported a certain ignorance with regard to the symptoms, which is perceived as a lack of awareness and respect by those affected, as *“You do not get any support from anyone.” (M/hO/nMh).* If, as a result of this, certain classifications or medical aids and appliances are not granted, the state of health or the economic situation may deteriorate.

*“But if you cannot afford Professor X [RB: head of a private clinic], then some consultants are appointed who do not know the illness. And then you do not get the contractual treatment.” (M/hO/nMh).*

Further barriers specifically resulting from the dualistic German health insurance system (statutory versus private) were reported. A lacking extent of services in statutory health insurance made access to adequate care difficult or impossible from the very start, according to some participants. This was especially true in case of financial burdens linked to fatigue symptoms and resulting social security issues.

*“If I could actually get the cold therapy paid for by my health insurance, because I have to pay for it myself from my 1,000 Euro pension. And that’s something that really stabilizes me. […] But if I do not get the support from the system, what should I do?” (F/hO/Mh).*

In addition, participants repeatedly experienced that medical practices or other healthcare facilities are hardly or not at all equipped to accommodate for the complexity of such symptoms. The fear of worsening symptoms, or even a crash in an overcrowded practice with long waiting times, leads some participants to postpone visits to the doctor or not attend check-ups (despite an increased risk of, e.g., cancer).

Some of the participants also brought up the position of science, and that the symptoms have not yet been researched enough (see #7 and #8 Table 3). They also expressed the need for answers in order to make their own experiences more tangible and explainable on the one hand, and to gain recognition on the other. This would also reduce the feeling of having to constantly justify oneself, as experienced by some.

*“How much money has been made available?! If you look at what is being researched for MS or in other areas. It’s so small.” (F/lO/nMh).*

As research progresses, perhaps the ongoing scientific controversy, which until now has also contributed to patients feeling they have been passed around and psychologized, will come to an end.

#### Intersectional discrimination

As indicated in the above sections, in some cases, there were differences in the participants’ experiences of perceived discrimination with regard to their social identities. For example, of the 19 participants interviewed, three reported no perceived discrimination, all of whom were men. Participants explained this on the grounds that they had withdrawn from their social environment due to their symptoms and/or arduous social interactions. In contrast, several female participants repeatedly reported sexist comments and stereotypes regarding their outer appearance and living circumstances.

*“The pain doctor I saw more or less told me this, well, you are a young woman and you must have psychological problems. And why do not you have a partner?” (F/hO/Mh).*

Such statements emerged as recurring and particularly stressful for the women and were experienced across different domains, ranging from close family members, friends, work colleagues to all kinds of medical staff. By attributing “*young”* and *“good looking”* (*F/lO/nMh*) to a women instead of treating her as patient who is to be taken seriously, participants reported intersecting age- and gender-based discrimination. Another statement in which intersectional discrimination became apparent was given by a female participant of Turkish origin. She was confronted with both culture-based and gender-based discrimination, as her sister expressed her disbelief: *“There is no such disease in Turkey.”* and *“But you look good. Why? Yes, why do not you do it!” (F/lO/Mh)*. In addition, the occupational status also had a certain influence. Participants with a higher occupational status were particularly reflective with regard to perceived discrimination at structural level and outlined possible approaches for improvement. The above-mentioned struggles applying for social welfare accompanied by financial worries, however, were exclusively expressed by participants with a low occupational status and lacking familial support, mostly single women – another example of intersectional discrimination.

*“Two and a half years ago I applied for benefits for reduction in earning capacity, I’ve now been assessed for the second time, it’s now going to the social court, or is already at the social court, yes, and I’m still waiting for a decision and I’m a bit desperate, because yes, the money is running out and that’s not a nice situation.” (F/lO/Mh).*

In contrast, married (by the majority male) participants were able to rely on their partner’s income and therefore were less affected by lengthy social security processes. Nevertheless, they complained about the non-utilization of helpful but costly medical services not covered by statutory health insurance.

## Discussion

### Summary and interpretation

To the best of our knowledge, this is the first qualitative study investigating perceived discrimination in fatigue as a persistent somatic symptom across different medical conditions and different domains also considering intersectional discrimination. For this purpose, a study sample was put together capturing a variety of social identities, symptom specifics, and experiences.

The qualitative data analysis resulted in eight main practices with several sub practices of perceived discrimination. The deductively selected domains in which discrimination was perceived by participants followed the ones elaborated by Thornicroft et al. (2022). Three out of nineteen participants stated no perceived discrimination, all of them were male. However, this was partly explained by the complete withdraw from the social environment due to their symptomatology. Across domains, communication appeared as a recurring theme. Sexist comments and reduction to outer appearance was reported especially by female participants. Also nonverbal communication was reported across domains, such as experiences of being smiled away, stared at or rejected. Participants particularly reported experiences of perceived discrimination in the medical setting and on structural level. The former referred to the communication with medical staff and the treatment itself, ranging from a lack of commitment and ignorance to refusal of treatment or even threat of drastic consequences regarding further medical and social care. Structural barriers appeared in light of the unaffordability of specific medical care not covered by statutory health insurance or bureaucratic processes in the social security system. These were more severe for (female) participants with lacking familial support. Work-related stigma was also reported, albeit not by all participants, but with drastic consequences for the participants’ work life and social security when superiors shirked their responsibility. Experiences of perceived discrimination in the social environment or in public seemed to be less frequent and less stressful for the participants. However, in light of the specific nature of fatigue symptoms (e.g., invisibility and lacking knowledge regarding etiology), quite repetitive patterns of discriminatory experiences appeared across domains which partly impacted or reinforced each other. For instance, general disbelief and psychologization could facilitate a misdiagnosis in medical settings which impedes necessary work-related adaptations and/or grant of social welfare, and simultaneously enhances denial of needed social support by family and friends. As discussed, intersectional discrimination became apparent in the interplay of discriminatory experiences due to fatigue symptoms with intersecting experiences of genderism, ageism, cultural racism, low occupational and/or familial status. Overall, participants pronounced perceived discrimination related to their fatigue symptoms more often than due to intersecting (stigmatized) social identities.

Already 25 years ago, research pointed out perceived discrimination in ME/CFS and persistent somatic symptoms (former ‘medically unexplained symptoms’) (Green et al., 1999; Kirmayer et al., 2004). Since then, research efforts have increased, lately due to the global and “mass-disabling” COVID-19 pandemic (Stelson et al., 2023). Echoing the findings of the present study, existing research underlined overarching perceived discrimination in the medical setting. This occurred explicitly and subtle with regard to disbelief and denial due to invisible and episodic symptoms as well as psychologization, diagnostic delay, treatment shortcoming due to a lack of knowledge, research on biomarkers and ignorance of patients’ experiences (Hussein et al., 2024; Ko et al., 2022; Pilkington et al., 2020; Treufeldt and Burton, 2024). Since fatigue symptoms are more often attributed to mental than somatoform causes, longstanding mental health stigma, not only among medical providers, seems plausible (Froehlich et al., 2021; Kornelsen et al., 2016; Lian and Lorem, 2017). Hence, attributed stereotypes correspond to the ones known from mental disorders, like being lazy or hypersensitive (König et al., 2024; Treufeldt and Burton, 2024). The question arises if someone with fatigue symptoms is justifiably ill, without own responsibility, and of unlikely recovery (Cheshire et al., 2021; Froehlich et al., 2021). In accordance with the present findings, existing evidence covered pressure to return to work full-time or dismissals due to sick leave resulting in great uncertainty and financial hardship of persons afflicted (Asbring and Närvänen, 2002; Brehon et al., 2023; Kornelsen et al., 2016). Studies also repeatedly stressed participants’ feelings of being unsupported, estranged from and judged by family and friends, which is often due to a lacking legitimite diagnosis for their symptoms as well as a lack of knowledge (Lacerda et al., 2019; Lian and Lorem, 2017; Moretti et al., 2022). Also, the ongoing debate about the misleading and labeling diagnostic terminology including terms like “post,” “chronic,” and “syndrome” was often criticized by study participants (Asbring and Närvänen, 2002; Byrne, 2022; Kornelsen et al., 2016). Further structural discrimination became apparent with regard to precarious financial situations, affordability of services, and service barriers due to necessary but lacking symptom-specific adaptations (Brehon et al., 2023; Stelson et al., 2023). German-speaking countries like Germany and Switzerland only recently came across the relevance and extent of health-related discrimination in fatigue with similar findings, despite country-specific health care services and social security system (Habermann-Horstmeier and Horstmeier, 2024; König et al., 2024). It must be noted, however, that perceived discrimination did not always occur to the same extent, since some studies also documented quite positive and satisfactory statements of participants regarding medical care and social support system (Asbring and Närvänen, 2002; Smyth et al., 2024).

Despite the assessed similarities of perceived stigma in fatigue, existing evidence mostly neglected to emphasize the intersection of stigmatized social identities and health-related discrimination or other barriers to health care, which is evident for several health outcomes like mental disorders or HIV (Henkel et al., 2008; Modani et al., 2024). Nevertheless, some studies investigated at least single social identities. Mostly, perceived discrimination, especially psychologization, denial of symptoms and unsupportive care, was more pronounced among females, black and ethnic minorities and younger, unemployed or heavier-weighted persons afflicted with ME/CFS or long COVID (Brehon et al., 2023; Green et al., 1999; Pilkington et al., 2020; Smyth et al., 2024). On the contrary, a mixed-methods study detected no gender- or age-based differences in perceived discrimination (König et al., 2024). Recently, two studies investigating long COVID revealed intersectional discrimination in older aged females and females from ethnic minority. The findings emphasized psychologization/gaslighting, dismissal of patients’ experiences and knowledge, social exclusion and nasty remarks due to a (culture-based) lack of understanding (Clutterbuck et al., 2024; Smyth et al., 2024). In contrast, investigating public stigma in fatigue, a study found significantly less public stigma toward female migrants with low occupational status, potentially reflecting a publicly expected and accepted ‘weak will’ of certain social identities (von dem Knesebeck and Barbek, 2023).

It is well studied that perceived discrimination is associated with a variety of negative as well as a few positive consequences regarding health outcomes and behaviors (Turan et al., 2019). Negative health outcomes can be, for instance, poorer physical and mental health, loss in daily functioning and less satisfaction with social roles and activities, increased distress, secondary depression, and suicidal thoughts (Froehlich et al., 2021; König et al., 2024; Kornelsen et al., 2016; Moretti et al., 2022). Health behavior and coping strategies can include concealment of symptoms and withdrawal from the healthcare system (Brehon et al., 2023; Clutterbuck et al., 2024; Hussein et al., 2024; Moretti et al., 2022), but also acceptance, self-advocacy, and activism (Asbring and Närvänen, 2002; Kornelsen et al., 2016; Turan et al., 2019). In some cases, enhanced medical mistrust and withdrawal exacerbate in the presence of financial constraints or felt discrimination due to, e.g., gender, ethnicity or body type, indicating intersectional discrimination. Due to intersecting systems of oppression associated with stigmatized identities, further worsening consequences occur, potentially widening existing health inequalities (Smyth et al., 2024; Stelson et al., 2023; Yang et al., 2014). Hence, it seems plausible that social, political, and economic expectations of illness, recovery, and the definition of ‘normality’ influence medical, organizational and structural behavior which in turn affects peoples’ health, social and economic wellbeing, and political power – often without acknowledging the perspective of those affected (Cheshire et al., 2021; Treufeldt et al., 2024; Turan et al., 2019).

### Strengths and limitations

Despite greatest efforts in the recruitment process, it is possible that the study sample does not fully reflect the heterogeneity of those affected and of their experiences. Further selection bias might have occurred, as patients with more strongly held views and openness to the topic under research are commonly more likely to participate. However, the diversity of the study sample can still be considered as a strength. The participants ranged from 30 to 69 years of age, included both females and males, different occupational backgrounds as well as persons with and without a history of migration. Although men with a history of migration and a high occupational status could be reached, it was not possible to include the same group with a low occupational status. In addition, it must be noted that specifically stigmatized social identities (for example, sexual and gender minorities, refugees or those who fall outside the welfare system) were not reached by this study. Nonetheless, the study sample was quite diverse regarding the onset of symptoms and diagnosis given. However, very severely affected persons had to withdraw their participation despite great interest due to their massively limited energy. The study is one of a few that looks at intersectional discrimination, adding new insights into stigma research in fatigue. It must be noted that intersectional discrimination can arise not only from the basis of different social identities, but also due to existing comorbidities, e.g., mental disorders. The latter were not considered in detail in the present analysis. Also not considered due to limited resources were consequences of perceived discrimination as well as strategies for reducing discrimination, both of which would have been of interest.

### Implications for research and practice

To deepen the understanding of drivers and mechanisms of (intersectional) discrimination, more research is needed on peoples’ reflections and explanations of their experiences of perceived discrimination, both in fatigue and related symptoms. Therefore, in addition to considering the perspective of those afflicted, it is also important to capture the perspectives of the other side, i.e., people from the respective medical, social or working environment. This would enable a more comprehensive understanding of the experience of discrimination. In addition, research is needed to further characterize the potential effects of shared social identities, e.g., how social support from people with similar intersectional social identities changes the way people react to and deal with stigma. Expanding this question facilitates in-depth understanding of why and how interventions should and could address intersectional discrimination (Turan et al., 2019). In this context, reference should be made to the Health Stigma and Discrimination Framework as a global, crosscutting framework on health-related, intersectional stigma for theory, research, and practice (Stangl et al., 2019). By applying this framework, future research can help to systematically identify interventions which are most appropriate in reducing intersectional fatigue-related stigma.

Thereby, the following, exemplary aspects should be considered to reduce perceived discrimination in fatigue as a persistent somatic symptom: In the medical setting, supporting person-centered care based on a bio-psycho-social approach with dedicated time, offering relevant in-house/outreach and virtual services. Also specialized, interprofessional care based on guideline recommendations, enhancing acceptance through empathy, is essential (Brehon et al., 2023; Habermann-Horstmeier and Horstmeier, 2024; Koczulla et al., 2024; Pilkington et al., 2020). In the work environment, an open-minded business culture, raising awareness and flexible workplace surroundings are needed (Stelson et al., 2023). Regarding the social environment, online communities, like on social media, and self-help groups for family and close friends seem promising to enhance knowledge, empathy and social inclusion (Berard and Smith, 2019; Froehlich et al., 2021). On the structural level, funding research on etiology and treatment options, finding a clinically useful, acceptable, and non-stigmatizing diagnostic terminology seems helpful. Also increasing knowledge and raising awareness through mass and social media and (medical) educational training regarding the symptomatology, and reducing bureaucratic barriers to enable low-threshold financial support seems appropriate (Berard and Smith, 2019; Hussein et al., 2024; Lian and Lorem, 2017). Additionally, findings on intersectional discrimination further emphasize the importance of structural interventions, e.g., regarding the male-dominated and dualistic approach in medicine or the cultural shaping of illness concepts (Treufeldt et al., 2024). Since multiple stigmatized social identities show increased distress as well as reduced resilience, psychosocial interventions are needed. This may also help to reduce health inequalities and enable long-lasting improvements in health (Mizock and Russinova, 2015; Turan et al., 2019).

## Conclusion

Most people affected by fatigue symptoms face discrimination across all kinds of intertwined domains, with great impact on health outcomes, health care and social protection. In particular, the lacking knowledge regarding fatigue-specific symptomatology reinforces social stigma, not only among medical providers but throughout all societal and institutional structures. By applying an intersectional stigma approach, intersectional discrimination, specific for certain stigmatized social identities (like female gender or low occupational status) became apparent. These need to be further researched and addressed in intervention strategies to reduce intersectional discrimination, increase resilience, and reduce health inequalities.

## Positionality statement

Recognizing that our identities may influence our approach to science (Roberts et al., 2020), we would like to provide the readers with some additional information on our backgrounds. The research team working on this study consisted of three researchers ranging from pre-doctoral researchers to professors. We represent different disciplinary backgrounds (health sciences, public health and medical sociology). With respect to gender, two researchers self-identified as women, one self-identified as man, and none of us has a history of migration. We would like to point out that none of us researchers are personally affected by fatigue symptoms. Experience with the symptom consists at most of contacts among friends and acquaintances.

We acknowledge the diversity of perspectives and influences that can shape our contribution to this research and have applied a reflexive approach in conducting it.

## Data Availability

The raw data supporting the conclusions of this article will be made available by the authors, without undue reservation.
